# Grocery Delivery to Support Individuals With Type 2 Diabetes: Protocol for a Pilot Quality Improvement Program

**DOI:** 10.2196/54043

**Published:** 2024-05-15

**Authors:** Lauren Oshman, Marika Waselewski, Rina Hisamatsu, Noa Kim, Larrea Young, Dina Hafez Griauzde, Tammy Chang

**Affiliations:** 1 Department of Family Medicine University of Michigan Ann Arbor, MI United States; 2 Institute for Healthcare Policy and Innovation University of Michigan Ann Arbor, MI United States; 3 Michigan Medicine Quality Department Ann Arbor, MI United States; 4 Veterans Affairs Ann Arbor Healthcare System Ann Arbor, MI United States

**Keywords:** T2D, type 2 diabetes, food insecurity, low carbohydrate, quality improvement, healthy eating, grocery, delivery program, diabetes, T2DM, type 2 diabetes mellitus, low-income, US, United States, adults, adult, low diet quality, carbohydrate, carbohydrates, glycemic control, nutrition education

## Abstract

**Background:**

People with low income are disproportionately affected by type 2 diabetes (T2D), and 17.6% of US adults with T2D experience food insecurity and low diet quality. Low-carbohydrate eating plans can improve glycemic control, promote weight loss, and are associated with improved cardiometabolic health and all-cause mortality. Little is known about supporting low-carbohydrate eating for people with T2D, although food-as-medicine interventions paired with nutrition education offer a promising solution.

**Objective:**

This program aims to support the initiation of dietary changes by using grocery delivery and low-carbohydrate education to increase the quality of low-carbohydrate nutrition among people with T2D and food insecurity.

**Methods:**

This program was a nonrandomized pilot conducted at 21 primary care practices in Michigan. Adults with T2D and food insecurity or low income were eligible to enroll. Patients were referred by primary care clinic staff. All participants received the 3-month program, which included monthly US $80 credits for healthy foods, free grocery delivery from Shipt, and low-carbohydrate nutrition education. Food credits were restricted to the purchase of healthy foods. Education materials, developed in collaboration with providers and patients, included print, digital, interactive web, and video formats. At enrollment, participants completed a survey including demographics, diabetes health, diet and physical activity, and diabetes management and knowledge. After the 3-month program, participants completed a survey with repeat assessments of diabetes health, diet and physical activity, and diabetes management and knowledge. Perspectives on participant experience and perceived program impact, food purchasing behaviors, and use of educational materials were also collected. Diabetes health information was supplemented with data from participant medical records. We plan to perform mixed methods analysis to assess program feasibility, acceptability, and impact. Primary quality improvement (QI) measures are the number of patients referred and enrolled, use of US $80 food credits, analysis of food purchasing behavior, participant experience with the program, and program costs. Secondary QI measures include changes in hemoglobin A_1c_, weight, medications, self-efficacy, diabetes and carbohydrate knowledge, and activity between baseline and follow-up.

**Results:**

This program started in October 2022. Data collection is expected to be concluded in June 2024. A total of 151 patients were referred to the program, and 83 (55%) were enrolled. The average age was 57 (SD 13; range 18-86) years, 72% (57/79) were female, 90% (70/78) were White, and 96% (74/77) were of non-Hispanic ethnicity. All participants successfully ordered grocery delivery during the program.

**Conclusions:**

This pilot QI program aimed to improve diet quality among people with T2D and food insecurity by using grocery delivery and low-carbohydrate nutrition education. Our findings may help inform the implementation of future QI programs and research studies on food-as-medicine interventions that include grocery delivery and education for people with T2D.

**International Registered Report Identifier (IRRID):**

DERR1-10.2196/54043

## Introduction

Over 12% of adults in Michigan have diagnosed type 1 or 2 diabetes, representing almost 1 million people. An additional 34.7% of adults, or 2.7 million people, have prediabetes [1]. Populations with low income are disproportionately affected by type 2 diabetes (T2D) [2]. Costs of diabetes related to complications including cardiovascular disease and kidney failure exceed US $7 billion dollars, making diabetes the most expensive chronic condition in the United States [3,4]. Medical nutrition therapy reduces cardiovascular complications of T2D, and low-carbohydrate diets (LCDs) are noted by the *American Diabetes Association Consensus Report on Nutrition Therapy* to improve glycemic control, promote weight loss, and improve lipid profiles. In addition, LCDs can reduce the need for hyperglycemic medications and promote diabetes remission, while LCDs emphasizing healthy plant-based foods are associated with decreased mortality [5-7].

Nearly 1 in 5 US adults with T2D experience food insecurity and low diet quality [8]. Drivers include low income, high cost of food, poor access to neighborhood grocery stores, lack of transportation, and lack of knowledge or time to prepare healthy foods. Food insecurity is associated with poorer diet quality, diabetes self-management skills, glycemic control, micro- and macrovascular complications, and increased health care use [9]. Evidence-based interventions to target food insecurity and low diet quality include food-as-medicine programs such as medically tailored meals, food pantry and produce prescriptions, diabetes self-management education, and participation in the federally funded Supplemental Nutrition Assistance Program (SNAP) and SNAP for Women, Infants, and Children (WIC) [9].

Adults with T2D are more inclined than the general population in Michigan to make changes in their diet and lifestyle [10]. This creates a window of opportunity to intervene and support behavior change that can positively impact their health and quality of life. Grocery delivery is a convenient and accessible intervention that addresses logistical barriers to obtaining healthy foods [11]. In 1 randomized controlled trial of patients with T2D, online grocery delivery with prefilled carts was associated with improved nutritional quality purchases when paired with healthy recipes [12]. Less is known about the implementation of online grocery delivery paired with healthy LCD education at scale in quality improvement (QI) initiatives for T2D. The purpose of this pilot QI program is to support the initiation of dietary changes by using grocery delivery and low-carbohydrate education to increase the presence of healthy, low-carbohydrate foods in the home.

## Methods

### Program Overview and Design

This pilot QI program, the Healthy Eating JUMPSTART (HEJ), was nonrandomized, and all participants received the program. The program consists of 2 main components, low-carbohydrate dietary education and grocery delivery of healthy foods. Participants were adults with T2D, and outcomes were measured by surveys, grocery purchasing data, and medical record (MR) review. The objective of this study is to assess the feasibility and acceptability of this program. The protocol follows the SQUIRE (Standards for Quality Improvement Reporting Excellence) checklist [13].

### Program Setting

The program was conducted at 21 primary care practices across Michigan belonging to physician organizations participating in the Michigan Collaborative for Type 2 Diabetes (MCT2D), a statewide Collaborative Quality Initiative focused on improving care for T2D in primary and specialty care practices across the state of Michigan. The team of researchers and staff involved in implementing this program included family and internal medicine physicians (LO, TC, and DG), a project manager (MW), a dietitian (RH), and 2 human-centered designers (NK and LY).

### Population and Eligibility Criteria

Program eligibility criteria were (1) aged ≥18 years; (2) diagnosis of T2D; (3) residence in a Shipt delivery zone; and (4) one of the following criteria for low income: Medicaid insurance status, positive screen for food insecurity, or self-reported earning <150% of federal poverty level based on household earnings and the number of people in the household. We planned to recruit a maximum of 150 individuals meeting program eligibility criteria.

Exclusion criteria were (1) type 1 diabetes; (2) active pregnancy or breastfeeding status; and (3) prescription for a sodium-glucose cotransporter-2 inhibitor (SGLT2i), as concomitant use of an SGLT2i and carbohydrate restriction can increase the risk of euglycemic ketoacidosis [6].

### Recruitment and Screening

Staff (case managers, social workers, dietitians, etc) at the participating primary care practices identified patients likely to meet program eligibility criteria. The physician or advanced practice provider for each potentially eligible patient then completed a signed attestation form, either paper or web based, confirming that the patient had a diagnosis of T2D, was interested in nutrition education, was not prescribed an SGLT2i medication, was willing to work with their primary care team to adjust insulin and sulfonylurea medication dosing as applicable, was not currently pregnant or breastfeeding, and agreed to be contacted by the program team. A program team member then reached out to the patient via email, phone call, or text to follow up on the referral and provide options to be screened for the program via a web-based form or phone call with program staff.

During screening, the home address was confirmed to verify the availability of Shipt delivery or pickup in their area. Financial status was assessed in a stepwise fashion using first a validated 2-question food insecurity screener, then Medicaid enrollment status, and lastly household income [14]. Team members then contacted patients via phone to review the program, confirm eligibility, answer patients’ questions or concerns, confirm contact information, and affirm their agreement to participate.

### Intervention

#### Overview

**Figure 1:**
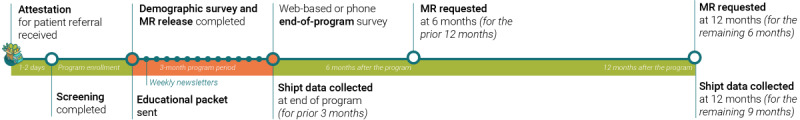
Timeline of participant enrollment from referral through completed data collection. MR: medical record.

The 3-month program consisted of healthy food delivery via Shipt and education to guide participants through adopting a lower carbohydrate eating plan. The timeline of the participant’s involvement in the program, including assessments, are outlined in Figure 1.

#### Grocery Delivery

The grocery delivery portion of the program provided free access to a grocery delivery subscription for 12 months plus 3 months of credits to purchase healthy foods that promote a low-carbohydrate eating plan to treat T2D. The program was delivered through the Shipt food delivery website [15]. Participants were provided with a US $80 Healthy Choice Allowance deposited on their Shipt account each month during the 3-month program period to be used to select from a limited menu of “healthy choice” food items. The Healthy Choice Allowance is an online credit that allows a customer to purchase from a restricted category of food, medicine, or other household items on the Shipt Marketplace. This amount of credit was chosen because (1) related healthy food pilots have noted changes in food purchasing behaviors with stipends around US $60 to US $100 per month [16,17]; (2) it is in line with current healthy food credits offered by insurance companies, which range from US $10 to US $300+ per month [18-21]; and (3) it allows for participants to place 2 delivery orders per month without a delivery fee (free on orders over US $35).

Participants could purchase a variety of healthy food options with their US $80 credit, including fruits, vegetables, proteins, dairy, healthy snacks, canned goods, and other staple foods (Textbox 1). Participants could not use the US $80 credit on items such as desserts, chips, pop, and candy but could purchase these items for delivery using their own funds. Healthy foods were not meant to supplant regular meals. Rather, they were intended to increase the prevalence of healthy, low-carbohydrate foods in the home.

**Examples of recommended healthy foods for program participants.**

**Vegetables**

- Kale, spinach, asparagus, mushrooms, tomato, cucumber, cauliflower, broccoli, peppers, green beans, and cabbage

**Fruits**

- Raspberries, blackberries, strawberries, plums, clementines, blueberries, peaches, and avocados

**Proteins**

- Chicken, eggs, pork, turkey, fish, tofu, and tempeh

**Other**

- Cheese, plain yogurt, sour cream, cottage cheese, nuts, and sunflower seeds

Participants had 2 options for placing food delivery orders depending on their preference: (1) participants could order independently using the Shipt website or (2) practice personnel could help participants place orders from the clinic. Program staff were available to support ordering as needed and provided an overview of using Shipt and the Healthy Choice Allowance to all participants during the onboarding process.

#### Low-Carbohydrate Education

Educational materials were developed by a registered dietitian (RH), physicians with expertise in low-carbohydrate eating plans for T2D who care for patients with diabetes (LO and DHG), and human-centered designers (LY and NK). Low-carbohydrate eating in the education intervention was defined as 50 to 130 g of total carbohydrates per day. Participants received a set of educational materials to teach them how to initiate and adhere to an LCD plan. These materials are available online and include education about the benefits of an LCD, meal planning strategies with a 7-day sample meal plan, grocery lists, nutrition label reading, and additional resources [22]. The resources were designed to introduce participants to the concept of low carbohydrate incrementally, starting with understanding the basics, then developing personalized low-carbohydrate goals, building meals, and tracking. Emphasis was placed on choosing high-quality sources of macronutrients and building balanced meals. A stepwise process for meal planning was described as consisting of diversity in nonstarchy vegetables, quality protein sources, healthy fats, and complex carbohydrates. Examples of food sources, recommendations for serving size, carbohydrate counting, and mindfulness tips were further conveyed in educational materials. Additional consideration was given to developing materials that would be accessible for individuals with low income including the creation of guides to eating low carbohydrates on a budget, categorization of recipes by price, developing recipes requiring limited appliances (eg, microwave friendly options), and highlighting weekly sales. Scientific evidence guiding the LCD plan was based on recommendations from the low-carbohydrate Diabetes Prevention Program diet program and aligned with the principles of the Healthy Low Carb Diet Score.

Participant educational resources were evaluated and revised in four stages: (1) automated assessment of Flesch-Kincaid Grade Level, (2) review by designers and clinical lead author, (3) live review by MCT2D affiliated health care providers, and (4) review by patients on the MCT2D Patient Advisory Board. With each iterative assessment, the team made revisions to improve the accessibility of the reading level, identify plain language words or phrases, provide accessible definitions, and reduce the number of words. Program materials were then adapted to be delivered in a variety of modalities including print, digital PDF, interactive web pages, and videos. At enrollment, all participants were mailed a packet containing a welcome booklet, which included resources on low-carbohydrate meal planning, a 3-month program calendar, and milestone stickers. They were then provided the link to the HEJ website and began receiving a weekly email newsletter designed to help introduce LCDs including low-carbohydrate recipes and blog posts. Because the team delivered new recipes and information via weekly newsletters, Shipt promotional sales and data regarding participant allergies and cultural or religious food preferences were incorporated into these weekly materials.

### Participant Retention

To maintain communication and engagement with participants throughout the program, the program team communicated with participants based on their preferred method. Phone calls, text messages, emails, and physical mailers were all used to share program information and to prompt participants to complete surveys. Email and text messaging were the default options. Phone calls were used only for individuals who selected it as their preferred form of communication or for individuals who were not responsive to other modes of communication. Participants were also contacted to notify them of unspent credits as they neared the end of each program month.

### Data Collection

#### Overview

Participants were enrolled in the program on a rolling basis between October 2022 and May 2023. Each participant’s enrollment started immediately after their screening and baseline survey and continued through a 3-month program period. The full program evaluation period continued through 12 months after enrollment. Participants completed an end-of-program survey either via phone, mail, or web-based form at the end of month 3. Data from Shipt were obtained in batches monthly during the program and at the conclusion of the evaluation period. MR data were requested from clinics in batches at month 6 and month 12 of the evaluation period.

#### Information Sources

All participants were asked to complete a set of baseline and end-of-program surveys during the program. Additional details on the assessments used are described below and listed in Table 1.

**Table 1:** Sources of pilot program data and timing of measurements for enrolled participants.

Measure			Data source		Measurement timing
**Sociodemographic information**					
	Age, race, ethnicity, gender identity, and highest level of education Food insecurity SNAP^a^ and WIC^b^ receipt Home food environment Primary food sources	Demographic survey		Baseline	
**Diabetes health information**					
	Height and weight	Demographic survey and medical record review		Throughout the evaluation period	
	HbA_1c_^c^ T2D^d^ diagnosis date Medications	Demographic survey and medical record review		Baseline and end of program	
**Diet and physical activity**					
	Physical activity	PAVS^e^ [23]		Baseline and end of program	
	Diet quality	ASA24^f^ [24]		Baseline and end of program	
	Self-efficacy for healthy eating	WEL-SF^g^ [25]		Baseline and end of program	
**Diabetes management and knowledge**					
	Diabetes and carbohydrate confidence	Likert question		Baseline and end of program	
	Sources of diabetes and nutrition information	Open-ended survey		Baseline	
	Challenges and benefits to low carbohydrate diet	Open-ended survey		Baseline and end of program	
	Diabetes and nutrition label knowledge	Selected questions from the Michigan Diabetes Research and Training Center’s Revised Diabetes Knowledge Test [26] and the AdultCarbQuiz [27]		Baseline and end of program	
**Perceived program impact**					
	Program helpfulness and perceived impact	Likert and open-ended survey		End of program	
	Diet changes and perceived impact	Open-ended survey		End of program	
	Likelihood to continue eating lower carb	Likert and open-ended survey		End of program	
**Program use data**					
	Food purchasing [15]	Type, quantity, and cost of foods purchased		Throughout the evaluation period	
	Website and newsletter analytics	Number of hits, unique users, and duration of use		Throughout the evaluation period	

^a^SNAP: Supplemental Nutrition Assistance Program.

^b^WIC: Women, Infants, and Children.

^c^HBA_1c_: hemoglobin A_1c_.

^d^T2D: type 2 diabetes.

^e^PAVS: Physical Activity Vital Sign.

^f^ASA24: Automated Self-Administered Dietary Assessment Tool.

^g^WEL-SF: weight efficacy lifestyle short form.

#### Sociodemographic Information

All sociodemographic information was collected upon enrollment, at baseline, via a secure web-based Qualtrics (Qualtrics International Inc) survey completed by participants, by printed and mailed survey, or via phone by the program team. This included age, self-reported race and ethnicity and gender identity, zip code, and highest level of education. Participants were additionally asked where they typically purchase food and groceries and whether any household members use SNAP and WIC. A modified version of the home food environment assessment, which measures the availability of the 13 major food categories in the home, was used to assess baseline food purchase patterns in the home [28]. Individual and household members’ food allergies and cultural or religious food preferences were additionally collected.

#### Diabetes Health Information

At baseline, participants reported their date of diagnosis of T2D and most recent HbA_1c_ (hemoglobin A_1c_) as well as a current list of medications and doses for the treatment of glycemia and other medications for diabetes such as statin medications. HbA_1c_ and medication changes were additionally self-reported by participants at the end of the program. Self-reported height and weight were also collected at baseline.

Diabetes health information was additionally collected through MR review. Participants signed an MR release form at enrollment via the University of Michigan’s online SignNow system or via a physical mailed copy permitting the release of MRs spanning 6 months before enrollment through 1 year after enrollment. MRs were then requested, in batches, from each participant’s diabetes care sites at 6 months and 12 months after enrollment. Study team members abstracted diabetes-relevant laboratory results (HbA_1c_, blood glucose levels, complete blood count, urine studies, liver or renal studies, and lipid results), vital signs (weight, height, blood pressure, and pulse), and medications prescribed during the entire 18-month period.

#### Diet and Physical Activity

Baseline and end-of-program physical activity and diet quality were assessed using the 2-question validated Physical Activity Vital Sign and the Automated Self-Administered Dietary Assessment Tool, respectively [29,30]. Self-efficacy for healthy eating was collected using the 8-question validated Weight Efficacy Lifestyle Short Form both at baseline and end of the program as well [25,31].

#### Diabetes Management and Knowledge

Participants completed a Likert scale regarding their confidence in diabetes management as well as a variety of different questions about their current sources of information about following a diabetes-friendly diet, whether they had worked with a nutritionist or dietitian, as well as their hopes for how the program would benefit them and what they anticipated may be challenging about following a low carbohydrate eating plan. Additional questions on diabetes and carbohydrate knowledge were also selected from the Michigan Diabetes Research and Training Center’s Revised Diabetes Knowledge Test and the AdultCarbQuiz [26,27].

#### Perceived Program Impact

Participants completed a web-based or phone-based survey at the end of the 3-month program. Participant’s experience with grocery delivery and LCDs, the impact of delivery on their diet, and the perceived impact on their T2D management and overall health were assessed. Experiences with educational materials were assessed iteratively and through multiple channels, using (1) a voluntary web-based survey 1 month into participation and (2) voluntary web-based or phone surveys with participants at the end of the program. Survey methods elicited both opinions of educational materials and understanding of which materials they used. These triangulated sources of feedback allowed the program to improve the content and delivery of supplemental educational materials iteratively. Descriptions reported in this paper reflect the final iteration of the program.

#### Program Use Data

With participant consent, obtained through the same methods as the MR release above, Shipt provided access to details on groceries purchased during the 3-month program period as well as the 1-year follow-up period in which participants retained free account access as part of the program. Data include the type, quantities, and costs of foods ordered by participants during the evaluation period.

Quantitative evaluation of program materials was conducted through the use of website and email marketing platform analytics. Analytics collected include link clicks from the emailed newsletters, detailed usage of each portion of the website, and unique users accessing information. Bounce rates were also assessed to evaluate sustained engagement with various pages of the website.

### Governance

This program was conducted as a collaboration between 3 Collaborative Quality Initiatives with overlapping missions related to healthy eating and lifestyle for Michiganders with T2D and low income: Healthy Behavior Optimization for Michigan, Michigan Social Health Interventions to Eliminate Disparities, and the MCT2D.

### Ethical Considerations

This QI program was deemed to be exempt from Institutional Review Board review by the University of Michigan Medical School Institutional Review Board. All participants verbally affirmed their interest in the program before enrolling. Participants were prompted to separately sign medical and Shipt record releases for data collection. No incentives were included for participating.

Participant data were collected via secure university-approved iterations of Qualtrics or REDCap (Research Electronic Data Capture; Vanderbilt University), phone calls, and printed surveys [32,33]. Nutrition survey data were collected via the National Cancer Institute–sponsored Automated Self-Administered Dietary Assessment Tool and did not contain any participant identifiers during collection. All data are stored in the Michigan Medicine SharePoint build, which is approved for personal health information storage and is only accessible by members of the program team [34]. Program IDs were assigned to all participants to allow for the deidentification of data for analysis to protect participant privacy.

### Primary QI Measures

The primary aim of this pilot QI program was to deliver a low-carbohydrate grocery delivery and educational program to people with T2D and low income or food insecurity. Therefore, the primary outcome measures were planned to evaluate the feasibility and acceptability of this program by participants with the following quantitative measures:

- Number of patients referred by each participating clinic for enrollment
- Number of patients enrolled in the program
- Use of the US $80 monthly credit
- Food choices and food ordering behavior with the US $80 credit and additional foods purchased by the participants during the 3-month program period, specifically assessing low carbohydrate food item purchase
- Participants reported experience with the program including the use of grocery delivery and educational materials
- Program costs include the cost of grocery delivery, food credits, and educational materials

### Secondary QI Measures

The secondary aims of this pilot QI program were to preliminarily assess the impact of the grocery delivery and educational program on a variety of diabetes control metrics. Clinical measures (weight and HbA_1c_) will be evaluated at follow-up time points (3 months, 6 months, etc) up to 12 months after enrollment, as available. The secondary QI measures are as follows:

- Percent change in HbA_1c_ between baseline and follow-up
- Percent change in weight between baseline and follow-up
- Percent of patients with diabetes control at baseline, defined as HbA_1c_<8, compared with follow-up time points
- Changes in diabetes medication between baseline and follow-up
- Changes in self-reported Weight Efficacy Lifestyle Short Form measured between baseline and 3-month follow-up
- Changes in diabetes and carbohydrate knowledge between baseline and 3-month follow-up
- Changes in Physical Activity Vital Sign results between baseline and 3-month follow-up

### Sample Size

Our sample size was determined based on participant flow, budgetary constraints, and published guidelines for designing feasibility pilot studies [35]. The sample was selected to provide sufficient data regarding the pilot program to understand methods and procedures to inform the potential dissemination of the program in a larger setting. Specifically, we planned to understand whether we could feasibly identify patients with T2D and low-income or food insecurity from primary care practices, enroll and retain them in the program, and deliver the program effectively to help participants participate in low-carbohydrate eating.

### Data Analysis

A mixed methods approach was used for data analysis. For quantitative results, we performed descriptive statistical analysis for baseline survey response data, including demographic characteristics and self-efficacy. For all continuous outcomes, we calculated median change and IQRs from baseline to 6 months and 12 months. Summary statistics will be calculated for all program experience data collected at the end-of-program survey. We will compare baseline and follow-up data points from closed-ended responses related to self-efficacy and diabetes knowledge (multiple choice, scales, dichotomous, and categorical) using standard quantitative techniques including *χ*^2^ tests for categorical data and 2-tailed *t* tests for continuous data (α=.05 for significance). These same methods will be applied to clinical metrics being assessed as secondary measures.

Brief text responses to open-ended questions from the end-of-program survey will be analyzed using standard qualitative techniques including content and thematic analysis [36-39]. Further, 2 investigators will review all responses, create memos of major concepts present, and iteratively develop a codebook. Responses will be coded, and major themes and conclusions determined by consensus conference. The themes identified from these responses will describe participants’ satisfaction or dissatisfaction with delivery, the perceived impact of food delivery on their health and diet, and other challenges or barriers to healthy behaviors managing T2D. These quantitative and qualitative data will then be merged to provide context and will be used in dissemination to tell the participants’ perspectives.

## Results

**Figure 2:**
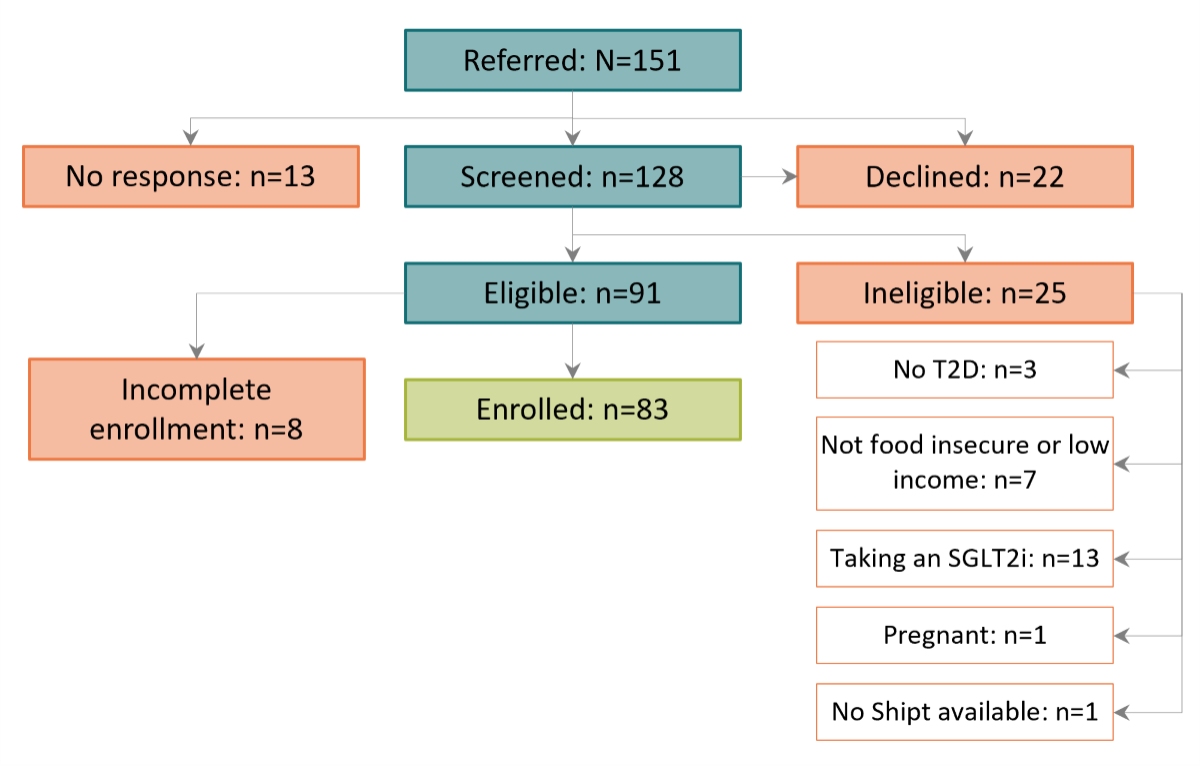
CONSORT diagram for patients referred to the program between October 2022 to May 2023. CONSORT: Consolidated Standards of Reporting Trials; SGLT2i: sodium-glucose cotransporter-2 inhibitor; T2D: type 2 diabetes.

During the enrollment period (October 2022 through May 2023), a total of 151 patients were referred to the program from the 21 participating practices, and we enrolled 83 (55%) participants (Figure 2). The 3-month program period has been completed for all enrolled participants as of September 2023. Data collection will conclude in May 2024 and results are expected to be published in 2025.

Enrolled participants were on average aged 57 (SD 13) years, with a range from 18 to 86 years (Table 2). They were predominantly female-identifying (57/79, 72%), White (70/78, 90%), and non-Hispanic (74/77, 96%). Participants most commonly had completed a high school education or less (35/79, 46%) or some college or technical school (25/79, 33%). During enrollment, 81% (67/83) of participants screened positive for food insecurity, while 14% (12/83) qualified based on Medicaid enrollment and 5% (4/83) based on income level. All participants were successfully able to order groceries during the program period and an example of the foods ordered with the US $80 monthly credit can be seen below in Table 3.

**Table 2:** Demographic characteristics for program participants enrolled between October 2022 to May 2023.

Characteristics			Values^a^
Age (years), mean (SD)			56.6 (13.5)
**Gender (n=79), n (%)**			
	Female	57 (72)	
	Male	22 (28)	
**Race (n=78), n (%)**			
	Black	4 (5)	
	Mixed race	1 (1)	
	Native American	1 (1)	
	Other	2 (3)	
	White	70 (90)	
**Hispanic (n=77), n (%)**			
	Yes	3 (4)	
	No	74 (96)	
**Education level (n=79), n (%)**			
	High school graduate or less	35 (46)	
	Some college or technical school	25 (33)	
	Associate’s or technical degree	8 (11)	
	Bachelor’s degree or higher	11 (14)	
**Eligibility criteria met (n=83), n (%)**			
	Food insecurity screen positive	67 (81)	
	Medicaid eligibility or enrollment	12 (14)	
	<150% of FPL^b^	4 (5)	

^a^Not all questions were answered by all participants, resulting in different N values for different characteristics.

^b^FPL: federal poverty level.

**Table 3:** Example monthly purchasing for a program participant.

Product name	Product price (US $)	Product quantity	Product total (US $)
Boneless and skinless tilapia fillets	6.39	1	6.39
Broccoli and cheese sauce	2.39	1	2.39
Cut okra	2.09	1	2.09
Frozen fruit bars variety pack, no sugar added	5.29	1	5.29
Grade A eggs large	3.99	1	3.99
Lemons	0.79	3	2.37
Mozzarella cheese and beef salami sticks combo pack	5.79	1	5.79
Pork sausage patties	4.09	1	4.09
Purified bottled drinking water	3.49	1	3.49
Purified drinking water	1.49	1	1.49
Roasted red potato in garlic sauce	3.29	1	3.29
Seasoned black beans	1.59	4	6.36
Seasoned Southern-style blackeye peas	1.79	1	1.79
Seasoned Southern-style collard greens	3.09	1	3.09
Tail-on medium peeled and deveined cooked shrimps	10.49	1	10.49
Wild-caught boneless and skinless Pacific cod fillets	6.99	1	6.99
Zespri sun gold kiwi	5.79	2	11.58

## Discussion

### Principal Findings

This protocol describes a pilot QI program to evaluate whether the implementation of the HEJ, a grocery delivery and healthy LCD education program, is feasible and acceptable for patients with T2D in primary care practices. Preliminary feasibility data demonstrate that primary care practices were able to refer appropriate patients with food insecurity and T2D to the HEJ and that 55% (83/151) of referred patients were able to enroll in the program. All enrolled participants successfully placed grocery orders and engaged with the program further providing evidence to support the feasibility of this program. The final end-of-program data analysis will focus on program evaluation, including participant knowledge and skill acquisition, and diabetes-specific outcomes to inform larger-scale implementation of similar programs.

Medical nutrition therapy and adherence to LCDs are evidence-based interventions associated with numerous benefits in T2D. We anticipate a positive change in the diets of program participants by making low-carbohydrate foods more accessible and convenient through the use of grocery delivery and healthy food credits. Long-term findings can help inform on whether anticipated dietary changes result in sustained change. Our results may also provide insight into the implementation of similar programs in the primary care setting to address food insecurity as a social determinant of health with the goal to improve T2D care and reduce complications and cardiovascular mortality.

The strengths of this pilot QI program include the simplicity of program design, ease of implementation in primary care clinics, and its relatively low cost. This program was additionally developed in the context of recent changes to allow SNAP food benefits to be used when shopping online. Its results may therefore help inform the implementation of larger-scale programs using online shopping and grocery delivery to reduce barriers to healthy eating for people with food insecurity and other associated social determinants of health [40]. The use of human-centered design to incorporate participant and provider feedback throughout the development of educational materials and program structure helped the team engage with individuals across the state. Further qualitative experience data from clinic staff and participants will provide richer detail into barriers and facilitators of the success of the program.

### Limitations

Limitations of this program include the inherent limitation to Shipt delivery zones, which does not fully encompass all patients with T2D in Michigan. Detailed evaluation of clinical impact as well as isolating the impact of the educational materials, the healthy food credits, and the grocery delivery components are not feasible in this iteration of the program as it was pragmatically implemented as a QI program. Changes in sociodemographic information and sources of diabetes information may impact participant outcomes but were not re-evaluated beyond baseline measurement. Our pilot participants were also overwhelmingly White and female, and further research should focus on understanding food insecurity among a more diverse population to ensure that larger-scale programs to improve health equity in T2D care reach communities most in need. Lastly, Shipt purchasing behaviors may not reflect the overall food purchased in the home as participants were also expected to obtain food from other sources.

### Conclusions

This pilot QI program aims to improve diet quality among people with T2D and food insecurity by using grocery delivery and low-carbohydrate nutrition education. Future research should more critically evaluate the impact on clinical outcomes and implementation costs in more rigorous randomized clinical trials. Our findings may help inform the implementation of future QI programs and research studies on food-as-medicine interventions that include grocery delivery and education.

## Abbreviations

HbA_1c_: hemoglobin A_1c_
HEJ: Healthy Eating JUMPSTART
LCD: low-carbohydrate diet
MCT2D: Michigan Collaborative for Type 2 Diabetes
MR: medical record
QI: quality improvement
REDCap: Research Electronic Data Capture
SGLT2i: sodium-glucose cotransporter-2 inhibitor
SNAP: Supplemental Nutrition Assistance Program
SQUIRE: Standards for Quality Improvement Reporting Excellence
T2D: type 2 diabetes
WIC: Women, Infants, and Children

## Data Availability

The data sets generated or analyzed during this study are available from the corresponding author upon request.
